# The first-person effect. A reconsideration of two meta-analyses

**DOI:** 10.1371/journal.pone.0311155

**Published:** 2024-12-11

**Authors:** Klaus Moser, Karsten I. Paul, Roman Soucek, Anett Eskofier, Nathalie Galais

**Affiliations:** Universität Erlangen-Nürnberg, Nuremberg, Germany; St John’s University, UNITED STATES OF AMERICA

## Abstract

The third-person effect describes a tendency to estimate the influence of mass communication on others (“third persons”) as being stronger than on oneself and this has been well documented in previous research. Though a first-person effect has also been postulated for desirable mass communication messages (for ex. non-profit advertisements or public service announcements (PSAs)), for which reporting more influenceability of the self as compared to others should be a means to self-enhance, it has not been found in the two named meta-analyses. One cause might have been ambiguities in the meaning of “impact” of desirable messages. For ex., whereas the content of the message might intend a desirable impact (for ex. a plead against violence), it can collide with low preferences for the respective message context (for ex. rap music) and thus a reported low impact of the message can result. We assume that this ambiguity with respect to the function of messages is considerably lower if only advertisements are considered because they have one main function: persuasion. We thus present reanalyses of data from two meta-analyses though restricted to studies on the impact of advertising. With data from the first meta-analysis, we not only replicate the well-known third-person-effect for undesirable messages (*d* = .83; 95%CI [0.72, 0.94]; *k* = 27), but we also find a first-person-effect for desirable messages (*d* = -.47; 95%CI [-0.70, -0.24]; *k* = 7). From the studies included in the second meta-analysis, we reanalyzed the results of studies with PSAs (all socially desirable messages). After the exclusion of some studies due to methodological problems, we find a first-person-effect for PSAs (*d* = -.16; 95%CI [-0.27, -0.04]; *k* = 33). Thus, contrary to the conclusions of both meta-analyses, we confirm the existence of a reliable first-person-effect. Replicability of meta-analytic results, the necessity to exclude studies due to methodological problems, and the meaning of “impact of socially desirable messages” are briefly discussed.

## Introduction

Mass communication is an important issue in such diverse settings as advertising, politics, education, public health, or religion. People not only *react* toward mass communication but also know that they are targets, and thus they often *judge* the effects of mass communication both on themselves and on other people. For example, judgments of marketers can be the basis by which to estimate the effectiveness of a commercial on consumers, or parents judge the probable impact of advertising on their children and then decide whether they allow their children to watch ads on television. Likewise, for consumers it is relevant because they might estimate the effect of advertising on others and if they assume the ad to be effective and the respective product is scarce, they might tend to hasten the decision to buy this product themselves. However, evidence that estimates on the impact of mass communication might be biased comes from research on one prominent effect, what Davison [1] named the "third person effect": "… people will tend to overestimate the influence that mass communications have on the attitudes and behaviors of others" (p. 3). In the following decades, an impressive number of studies has been conducted [2] on this phenomenon, which has also been relabeled as “third person perception”. This has been done because the mentioned definition describes a perceptual phenomenon, i.e., people are asked to *estimate* the impact of mass communication. We will, in line with [2], use the more “traditional” term.

Subsequent research proposed a motivational tendency as one important explanation for the third-person effect: It is socially desirable and positively valued that oneself is less influenceable than other persons are. In other words, the third-person effect is the result of self-enhancement [3, 4]. One important implication of this explanatory account is as follows: If there exists a condition under which it is normatively appropriate (“desirable”) to be influenceable, people should tend to estimate the impact on themselves as being stronger when compared to the impact on others. Consider for example an advertisement to spend more time with one’s children. Under this condition, it is socially desirable to be influenceable and to “… acknowledge receptivity to messages designed to impart positive social values” [5, p. 282]. Thus, respondents can be expected to estimate the impact on themselves as being stronger than on other people, i.e., a first-person effect (FPE) should result.

In their comprehensive meta-analysis [5], analyzed 372 effect sizes on the third-person-effect. Whereas for undesirable and ambiguous messages, the mean effect sizes were large and significant (*d* = .86, respectively, *d* = .65) with a positive sign indicating a third-person effect, for desirable messages the effect was negative (*d* = -.17). However, this did not mean that a first-person effect emerged for desirable messages. More precisely, under the condition that messages were desirable, the confidence interval of the mean effect size included zero. In the following, we explain why this lack of a first-person effect might be a result of ambiguities in the functions of “desirable messages” as available in the sample of studies analyzed.

Research on the perceived impact of media messages on the self and on others asks participants about the effects of media in general, TV news, political campaigns, advertising, pornography, or campaigns for organ donations, to name just a few examples. Analyzing the moderating effect of the desirability of the messages on the difference between perceived impact on the self and on others has to take into account that messages used in the studies that were meta-analyzed can differ in an important number of dimensions. In particular, the respective messages and media have different functions (e.g., entertainment vs. information vs. persuasion). This heterogeneity could be confounded with the desirability of the messages and thus their perceived impact, and can be an explanation for the missing first-person-effect in the Sun et al. meta-analysis. For example, newspapers or TV news (for ex. [6]) reporting on negative effects of binge drinking or smoking, though also understandable as a pleading for socially desirable behaviors, might primarily be perceived by research participants as fulfilling or failing to perform their core functions: informing and entertaining their audiences. Thus, a request to estimate the impact of this kind of messages can be understood in various ways, for example whether recipients “understood” the messages or whether they saw them as being entertaining. As another example, though prosocial rap-lyrics might be considered as “socially desirable” with respect to the messages (cf. [7]), it might at the same time not fit with the preferred music style of the recipients probably resulting in a low perceived impact even though the respondents agree with the content of the socially desirable message. As a preliminary reaction to the problem that messages can have multiple functions and thus create ambiguity in study participants that are asked to rate their impact, it might be advisable to restrict the analysis to messages that can have desirable or undesirable effects *and* have one core function. We consider *advertisements* as being particularly appropriate because they have such a core common function, *persuasion*, and their effects can be considered as being “socially desirable” (for ex. promote citizenship) or “socially undesirable” (for ex. make people buy expensive products which they might not be able to afford). We therefore conducted a meta-analysis based on a reanalysis of the data [5], though with an important modification: we restrict our reanalysis to studies on the perceived effects of *advertising*.

## Study 1: A partial reconsideration of Sun et al. (2008)

### Method

We used the data file generously made available by Ye Sun, which was the basis of the meta-analysis [5] incorporating 372 effect sizes. We selected all studies with messages related to advertising and we used the coding from the data file with respect to the social desirability of the message content: (1) low (e.g. commercial ads, political ads), (2) high (mainly ads for social causes), (3) ambiguous. The supporting information explains why we excluded two studies. When several effect sizes for the same sample were reported (for example separate effect sizes for “in-group others” and “out-group others”), an average effect was computed in order to avoid statistical dependencies among the effect sizes used in the meta-analytic computations. With few exceptions, information on effect sizes and sample sizes were used as documented in the database. The exceptions pertained to three effect sizes where the sign had to be reversed because a careful check of the respective primary studies revealed that coding errors had occurred in the original database. We used a random effects model meta-analysis, because we considered the data to be heterogeneous [8]. Cohen’s *d* effect sizes were taken from [5]. We also conducted a moderator test for desirability using a random effects model applying the method of moments, as described in [9].

## Results

We identified 39 effect sizes in the database that are related to advertising. The overall effect was of medium size and positive, indicating a TPE (*d* = 0.56; 95%CI [0.39, 0.73]; *k* = 39; cf. Table 1). The moderating effect of desirability was highly significant (p < .001). As expected, the mean effect size for undesirable messages was large and positive, indicating a TPE (*d* = 0.83; 95%CI [0.72, 0.94]; *k* = 27). More importantly, a FPE emerged for desirable messages, with an effect of medium size (*d* = -0.47; 95%CI [-0.70, -0.24]; *k* = 7). For studies with message content of unclear or ambiguous character, a relatively weak TPE was found (*d* = 0.53; 95%CI [0.27, 0.79]; *k* = 5).

**Table 1:** Meta-analytic results of the Sun et al. (2008) database (Only advertising-studies).

	*Q*_*b*_	*p*	*N*	*k*	*d*	*95% CI*	*Q*_*W*_	*p*
Total sample			6271	39	0.56	[0.39, 0.73]	1087.99	< .0001
Desirability	99.39	< .0001						
Low			4711	27	0.83	[0.72, 0.94]	42.16	.0237
High			736	7	-0.47	[-0.70, -0.24]	3.93	.6857
Ambiguous			824	5	0.53	[0.27, 0.79]	3.30	.5087

*Notes*. *k* = Number of effect sizes; *N* = Sample size; *d* = Average meta-analytic effect size (standardized mean difference); CI = 95% confidence interval for *d*; *p* = Significance level for *d*; Q = Heterogeneity statistic; Q_b_ = Heterogeneity between groups; Q_w_ = Heterogeneity within groups.

Thus, a reanalysis of the dataset [5] yielded a strong TPE for undesirable advertising messages. However, and more importantly, there was also a considerable mean FPE for desirable advertising messages.

## Discussion

If self-enhancement explains the third-person effect (TPE), then it should reverse into a first-person-effect (FPE) in case of socially desirable persuasive intentions of communication. However, previous research found no consistent evidence for this reversal [5] and concluded that this might be “… in part due to a much smaller number of effect sizes in the extant literature” [5, p. 294]. We proposed an alternative explanation for difficulties in finding a generalizable first-person effect: The effects of message desirability on the perceived impact of oneself compared to on others might have been underestimated in previous research due to ambiguities in the functions of a part of the “desirable messages”. We therefore conducted a reanalysis of a dataset from a previous meta-analysis though restricted to messages with a clear function, i.e., advertising messages [5]. In fact, whereas the size of the TPE for undesirable messages turned out to be comparable to previous research [5], an FPE emerged for desirable messages.

Our results should be treated with some reservations. First, the generality of the effectiveness measures (for ex. “influenceability” vs. “influenceability of behavior”) differs between the studies, making a one-to-one comparability of the effects of desirable vs. undesirable messages less than perfect. Second, we also included studies on political advertising, though, for example, the evaluation of negative political advertising as undesirable might also depend on the partisanship of the raters of the respective campaigns. Whereas these limitations are only of concern for the size of the TPE in general, another concern is that the results for desirable messages are based on only seven effect sizes. Thus, the question emerges whether this effect of desirable advertising messages would be replicable in more recent and additional studies. A partial answer could be found in a more recent meta-analysis [10], which included (as reported in [10]) 30 studies and 170 effect sizes on the perceived effects of public service announcements (PSAs). Of note, this meta-analysis reports an average *d* = -0.092 which again suggests that no generalizable first-person-effect for this kind of socially desirable advertising messages can be found. However, our partial reanalysis of the first dataset [5] also suggested that it might be recommendable to scrutinize whether it was appropriate to include each primary research study (see again the supporting information).

## Study 2: A partial reconsideration of Eisend (2017)

### Method

We used the data file generously made available by Martin Eisend, which was the basis of the meta-analysis of [10]. We selected all studies with PSAs and we used the coding from the data file with respect to the existence of a PSA. A preliminary analysis resulted in the observation that there exist differences in opinions between [10] and us in how a part of the studies should be coded and which criteria for inclusion of studies should be used. We therefore decided to carefully read all studies and rated them with respect to their appropriateness, in particular the message contents and the rating scales used. Again, when several effect sizes for the same sample were reported, an average effect was computed in order to avoid statistical dependencies among the effect sizes used in the meta-analytic computations. Information on sample sizes were used as documented in the database. We again adopted a random effects model meta-analysis of correlations as an appropriate perspective for our analysis, because we considered the data to be heterogeneous [8]. Cohen’s *d* effect sizes for change scores [11] were used in order to represent the first-/third-person perceptions reported in the primary studies. Hedges correction factor was used for deriving the unbiased effect sizes [12]. We used REML-estimation in SPSS (Version 28.0.1.1.).

### Results

**Fig 1:**
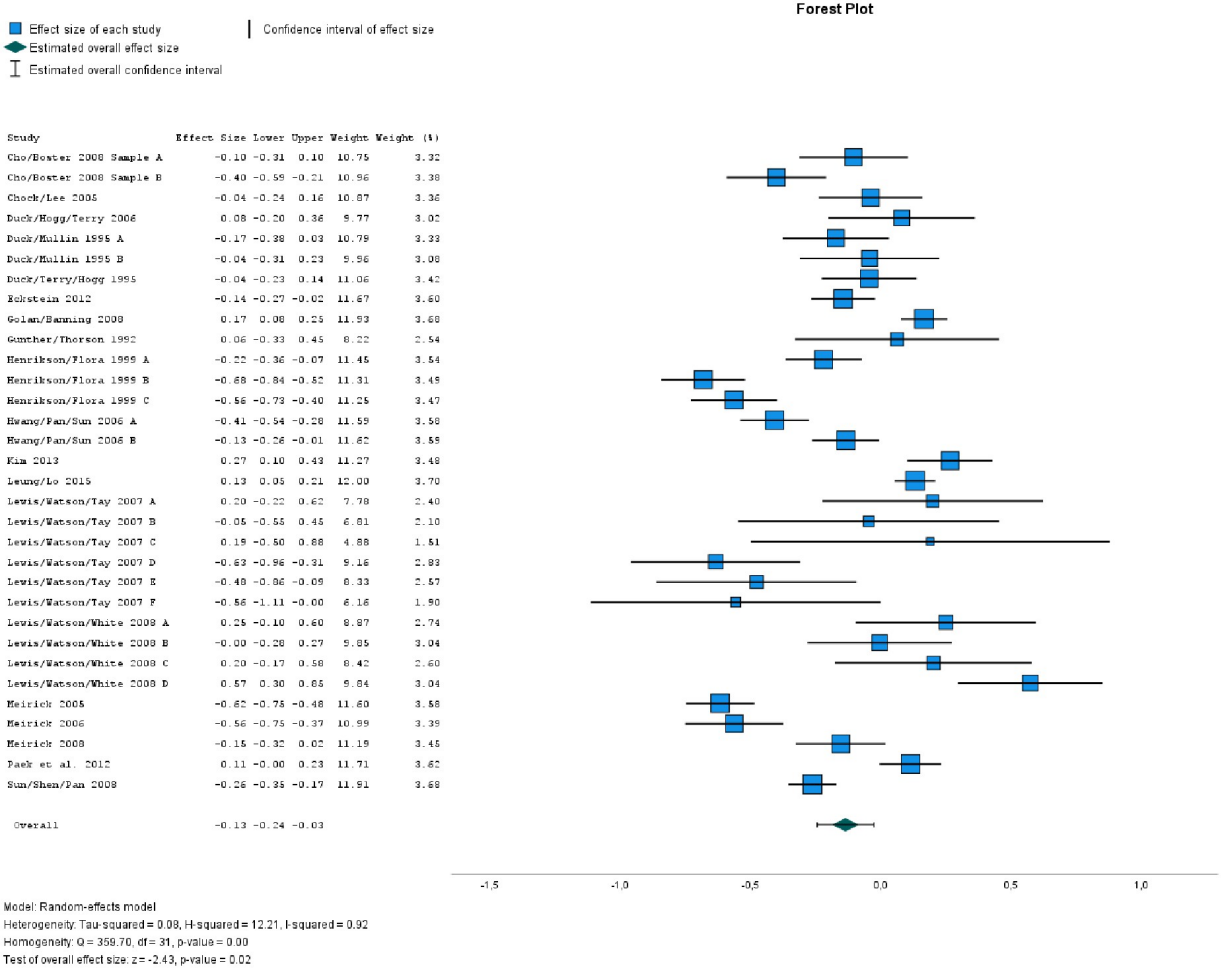
Effect sizes and forest plot for results of Study 2.

We started with the studies in the database that are related to PSAs. We then carefully read and repeatedly discussed the appropriateness to include the respective studies. The supporting information reports how we proceeded in detail and why we excluded some studies. The subsequent meta-analysis based on [13–32] yielded an FPE (*d* = -0.16; 95%CI [-0.27, -0.04]; *k* = 33; see Fig 1). In addition, Egger’s test for funnel plot asymmetry showed that the results are probably not affected by publication bias. Thus, the second estimate of the size of the FPE for a class of socially desirable messages (PSAs) based on a bigger number of studies again confirms the existence of a FPE, though with a smaller mean effect size than that suggested based on the first meta-analysis.

## General discussion

Estimating the effects of mass communication is important in areas such as commercial advertising, political communication, or public health programs. One often-researched phenomenon is the third-person effect [5], that is the tendency to perceive effects of mass communication as being stronger on others (“third persons”) than on the self. Though a first-person effect has also been postulated for desirable mass communication messages, for which reporting more influenceability of the self as compared to others should be a means to self-enhance, it has not been found in two meta-analyses [5, 10]. We presented partial reanalyses of the data from these two studies. We started with a meta-analysis [5] which is up to now one important standard of reference in research on the TPE. For example, recent research on both the FPE and the TPE tends to use this meta-analysis as a backup and comparison standard (for ex. [33, 34]). However, our focus was on advertising messages because this has a clear advantage with respect to one common main function of the messages analyzed: persuasion. We found a significant FPE. Another more recent meta-analysis [10] also had a focus on advertising. Again, we found an FPE for PSAs, the most prominent example of advertising messages that have an impact which is socially desirable. In sum, our results are consistent with the self-enhancement explanation of both the TPE *and* the FPE.

Of note, the most important difference between our analyses and the analyses presented in [5, 10] were our decisions to exclude a number of studies because they did not fulfill important conditions. For example, some studies used messages with a content that can be considered as socially desirable to be influenced by, however participants who would confirm an effect on themselves would also admit that their previous behavior has been far less than desirable (e.g., [35]). More generally, if people are asked to estimate an effect of a socially desirable persuasive message on themselves, they may be reluctant to confirm this for two reasons. First, after all, it is still a matter of conformity, that could in turn also provoke reactance [36]. And second, a potential effect might presuppose irrelevant conditions. As an example, participants in [13] watched four anti-drunk driving advertisements and were then asked to indicate the extent that ‘‘you yourself would be influenced” and ‘‘other drivers in general would be influenced by the advertisement”. One of these advertisements suggested that if a driver of a car has consumed alcohol, it is a good decision to let others (a companion) drive the car. This procedure suggests that “influenced” might mean to act in a comparable way, which however would be irrelevant for people who *generally* do not drink alcoholic beverages, and even for those who drive to parties by car but unaccompanied. Thus, specificity of action that is suggested by both the persuasive message in use and the stimuli presented to participants might be worth being more thoroughly analyzed in future research on the first-person-effect.

### Limitations

We decided to analyze advertisements because they are the most common example of mass communication in the Western world. In addition, they represent the range of more or less socially desirable messages because for ex. both for-profit and non-profit organizations use advertisements. However, advertisements are only one type of mass communication. In addition, the equation of non-profit organization and using advertisements with a socially desirable impact has its limits. For example, political advertising can also be considered non-profit advertising though a considerable proportion of citizens are not convinced that it is socially desirable to be persuadable by the respective activities [37]. In fact, the unequivocal desirability vs. undesirability of any message effectiveness can be debatable [10]. The desirability of a message could be evident for the members of a certain group (e.g., a political advertisement to vote for the leader of the group), but not for members of other groups (e.g., followers of an opposing party). As another example, though nowadays many people would agree that organic products should be preferred and thus buying them is desirable, this opinion is not shared among all individuals. Moreover, there can exist conflicts between injunctive and descriptive rules. For example, an injunctive rule in many countries in the world is to buy a ticket before entering a bus. However, the emerging descriptive rule might be that almost nobody seems to buy a ticket. Therefore, it is not really clear whether it is socially desirable from the respondent’s perspective to buy a ticket (because the prescriptive norm deviates from the descriptive norm), and it will therefore be an open issue whether for example a FPE would emerge concerning the estimated effect of a media campaign against fare beating. Finally, message desirability is related to the perceived credibility of the message and the integrity of the sender. For example, donations for children in need might be considered desirable behavior, however concerns that the money will be wasted on red tape or will be otherwise misused could lead to a skeptical view on the social desirability of a donation. In sum, the recipient has to believe that it is “really” socially desirable to report a respective message effect.

We were quite critical in our reevaluation of the primary studies used in the meta-analyses. However, we still decided to use almost all effects available even though we were concerned about the inclusion of some others of them besides those that we excluded. For example, one study used advertisements of an anti-smoking campaign [14]. However, the majority of the research participants were non-smokers, which makes it difficult to give an unequivocal interpretation on what participants mean when they estimate the effects of the advertisements on their smoking behavior. In case of doubt, we decided to accept that participants made reasonable estimates of the effects of a message on themselves and on others.

## Conclusions

With respect to more general implications, we consider three issues deserving particular attention. First, our analyses show that meta-analyses are not only an important starting point to guide subsequent research [38], but that they themselves should be treated like primary studies. This means that they should be in total or at least partially (as in our research) reproducible and that a critical review of the inclusion criteria of individual studies can generate valuable knowledge. We acknowledge that the authors of both meta-analyses shared their data. However, warnings against overestimating the importance of meta-analyses for a field of research [39] seem justified given the considerable amount of judgment calls that were obviously necessary. Finally, readers might have expected that the more recent meta-analysis [10] would also include the relevant studies of the earlier one [10]. We were surprised to find only a limited number of studies that were included in both meta-analyses.

Second, our focus on advertisements is an important contribution to the issue of comparability of messages that differ in social desirability. However, both for-profit advertisements and non-profit advertisements or PSAs often address more than one level of impact to persuade and thus they can differ with respect to the relative importance of these levels. Even if we assume that all advertisements want to persuade, it is important to recognize that the respective primary impact levels might be different. Asking whether advertisements have an “impact” or have “influence” could mean something different for for-profit ads compared to non-profit ads. This would even be true if the messages ask for a comparable amount of money from the recipients. For example, an advertised burger must be paid for, whereas a fixed amount of money is not necessary to spend in order to help refugees. In addition, the “worth” one gets for spending a comparable amount of money for a burger or a good deed obviously differs, and we assume that most people are aware of this difference. In addition, advertisements in the non-profit area sometimes primarily intend to increase *awareness* of an issue [40], most notably PSAs, whereas for-profit advertisements are more often directed at having effects on buying behaviors. Unfortunately, almost no study on the FPE included information on more than one impact level. Future research should therefore analyze the TPE as well as its reversal (a FPE) based on an explicit distinction of perceived impact levels. Third, and finally, self-enhancement is only one of several accounts proposed as explanations for the third-person effect. The current research intended to explain why a reversal of the TPE, which is consistent with the self-enhancement account, might not have been found in both previous meta-analyses. This does not mean that alternative accounts are less valid [41] or are even not able to predict a first-person effect for socially desirable messages.

## Supporting information

S1 Appendix Reasons for the exclusion of studies analyzed in Sun et al. (2008). (DOCX)

S2 Appendix Reasons for the exclusion of PSA-studies analyzed in Eisend (2017) and other remarks on computational issues. (DOCX)
