# Supplementary material for: The first-person effect. A reconsideration of two meta-analyses
Source: PLoS One. 2024 Dec 11;19(12):e0311155. doi: 10.1371/journal.pone.0311155 (PMC11633950; doi:10.1371/journal.pone.0311155)
Supplement: S1 Appendix — (DOCX) [file pone.0311155.s001.docx]

## Appendix 1: Reasons for the exclusion of studies analyzed in Sun et al. (2008)

1. We did not include David et al. (2004) and Hoorens and Ruiter (1996). In both studies, scales were used that created results not comparable to those of the other studies and were also inconsistent with the usual definition of a third-person effect. Hoorens and Ruiter (1996) used bipolar scales with a social comparison instruction, that is, participants were asked to indicate how much a message has an effect “… in comparison with the impact … on the average student …”. This means, that participants were asked to mentally compute the difference of the impact, whereas the usual approach is to collect the ratings of the estimated effect separately and then compute the difference. David et al. (2004) used bipolar scales with anchors describing opposing behavioral effects. For example, participants were asked to rate whether a message on alcohol consumption “strongly discourages drinking” or “strongly encourages drinking”. Since both ends of the scale represent strong message effects, the computation of mean scores across subjects to describe the strength of effects of a message (as required to compute a third-person-effect) has no clear interpretation.

**References**

David, P., Liu, K., & Myser, M. (2004). Methodological artifact or persistent bias? Testing the robustness of the third-person and reverse third-person effects for alcohol messages. *Communication Research*, *31*(2), 206-233. https://doi.org/10.1177/0093650203261513

Hoorens, V., & Ruiter, S. (1996). The optimal impact phenomenon: Beyond the third person effect. European Journal of Social Psychology, 26(4), 599–610. https://doi.org/10.1002/(SICI)1099-0992(199607)26:4<599::AID-EJSP773>3.0.CO;2-7
