# Supplementary material for: The first-person effect. A reconsideration of two meta-analyses
Source: PLoS One. 2024 Dec 11;19(12):e0311155. doi: 10.1371/journal.pone.0311155 (PMC11633950; doi:10.1371/journal.pone.0311155)
Supplement: S2 Appendix — (DOCX) [file pone.0311155.s002.docx]

## Appendix 2: Reasons for the exclusion of PSA-studies analyzed in Eisend (2017) and other remarks on computational issues

Eisend reports the inclusion of 30 studies. In the following, we explain why we excluded a number of them in our reanalysis. We also add some remarks where we decided to use the computations as utilized in Eisend (2017) or Sun et al. (2008).

*No study on the perceived effects of a message.* Anker (2007) was excluded for that reason.

*Implicit undesirable own behavior in the past.* Some studies ask participants whether the advertisement would make them change their behaviors in a *more appropriate* way. This however means that participants who agree would at the same time admit undesirable behaviors. This has been done in Chapin (1999a, b), Innes and Zeitz (1988), and Mackert et al. (2014).

*No study of a PSA.* Both Day et al. (2008) and Smith (2008) did not analyze the effects of a PSA.

*Multiple reporting problems.* The study of Gunther and Mundy (1993) contains three problems. First, it is not clear whether they were successful in designing PSAs. Second, they used scales with anchors allowing research participants to estimate reactions in opposing directions. A look at the data for “harmful” messages confirms that problem. For example, in three cases subjects indicated that they estimate the effects as being *stronger on themselves* than on others, though of course in the negative direction. According to Gunther and Mundy (1993), this is still evidence for a third-person effect. We disagree. Third, it is not possible to extract information on sample sizes for the specific effects and then calculate an appropriate effect size. The reason is that each research participant (n = 73 in total) received only a small part of the stimuli.

We also found that the Chock et al. (2007) study reports results that lead to contradictory estimates of the difference between effects on self vs. on others. On p. 628, there are two mean differences reported, *M* = 0.55 and *M* = 0.89. However, according to table 2, last row, the differences are even higher (1.13, 0.98, 0.96). Finally, according to table 4, last row, we can compute the total difference across all advertisements and weight the means by the respective sample sizes (*n* = 57; 58.8 % vs. *n* = 40; 41.2 %) which results in *M* = 0.08. Given such a range of estimates we decided to exclude this study.

*Inclusion of the Eisend (2017) data.* There are five other studies for which we were not able to locate all necessary data, but we decided to include them and use the estimates as reported in the Eisend-dataset: Duck et al., 2006, Duck and Mullin, 1995 (2 studies), Duck et al. (1995), Gunther and Thorsen (1992).

*Final caveat on a measurement ambiguity.* Meirick (2005) reports that people rate themselves more influenced than others by anti-smoking and anti-drunk-driving PSAs. However, the scales used allowed research participants to estimate reactions in opposing directions. More specifically, responses could range from „*Makes me/them much less likely to smoke/drive drunk*” to „*Makes me/them much more likely to smoke/drive drunk”*). This means that even an anti-smoking ad could have an effect such that it makes recipients to estimate an increase (!) of smoking. Though this would be an „effect” of the message, it is treated by the author as if the message has no effect, given that arithmetic means were computed. We assume that this is a minor problem and decided to keep the study. A similar problem concerns the Gunther and Thorsen (1992) study (scales allow research participants to estimate reactions in opposing directions). Again, we assume that this is a minor problem and decided to keep the study.

**References**

Anker, A. E. (2007). *Influencing intentions to donate blood: The use of threat and first-person effects.* Unpublished master’s thesis, State University of New York at Buffalo.

Chapin, J. (1999a). *Advertising vs. public service announcements: The role of message type in safer sex campaigns and third-person perception.* Paper presented at the annual meeting of the Association for Education in Journalism and Mass Communication, August, New Orleans, LA.

Chapin, J. (1999b). *Third-person perception, optimistic bias, safe-sex campaigns, and sexual risk-taking among minority “at-risk” youth.* Paper presented at the annual meeting of the Association for Education in Journalism and Mass Communication, August, New Orleans, LA.

Chock, T. M., Fox, J. R., Angelini, J. R., Lee, S., & Lang, A. (2007), Telling me quickly: How arousing fast-paced PSAs decrease self-other differences. *Communication Research, 34*(6), 618-636. https://doi.org/10.1177/0093650207307900

Day, A. G. (2008). Out of the living room and into the voting booth: An analysis of corporate public affairs advertising under the third-person effect. *American Behavioral Scientist, 52*(2), 243-260. https://doi.org/10.1177/0002764208321354.

Duck, J. M., Terry, D. J., & Hogg, M. A. (1995). The perceived influence of AIDS advertising: Third-person effects in the context of positive media content. *Basic and Applied Social Psychology*, *17*, 305-325. https://doi.org/10.1207/s15324834basp1703_2

Duck, J. M., & Mullin, B.-A. (1995). The perceived impact of the mass media: Reconsidering the third-person effect. *European Journal of Social Psychology, 25*(1), 77-93.

https://doi.org/10.1002/ejsp.2420250107

Duck, J. M., Hogg, M.A., & Terry, D. J. (2006). Social Identity and perceptions of media persuasion: Are we always less influenced than others? *Journal of Applied Social Psychology, 29*(9), 1879-1899. https://doi.org/10.1111/j.1559-1816.1999.tb00156.x

Eisend, M. (2017). The third-person effect in advertising: A meta-analysis. *Journal of Advertising*, *46*, 377-394. https://doi.org/10.1080/00913367.2017.1292481

Gunther, A. C., & Mundy, P. (1993). Biased optimism and the third-person effect. *Journalism Quarterly*, *70*(1), 58-67. https://doi.org/10.1177/107769909307000107

Gunther, A. C., & Thorsen, E. (1992). Perceived persuasive effects of product commercials and public service announcements: Third-person effects in new domains. *Communication Research, 19*(5), 574-596. https://doi.org/10.1177/009365092019005002

Innes, J. M., & Zeitz, H. (1988). The public’s view of the impact of mass media: A test of the ‘third person‘ effect. *European Journal of Social Psychology, 18*, 457-463. https://doi.org/10.1002/ejsp.2420180507

Mackert, M., Lazard, A., Champlin, S., Liang, M.-C., Mabry, A., Stroever, S., Guadagno, M., & Watkins, L. (2014). Take time, Safe lives. Clean hands protect: A comparison of two hand hygiene health promotion posters. *American Journal of Infection Control, 42*(5), 530-532. https://doi.org/10.1016/j.ajic.2014.01.017

Meirick, P. C. (2005). Rethinking the target corollary: The effects of social distance, perceived exposure and perceived predispositions on first- and third-person perceptions. *Communication Research*, *32*(6), 822–843. https://doi.org/10.1177/0093650205281059

Smith, P. J. (2008). A relationship-based approach to understanding the role of race cues in third-person perception, unpublished master’s thesis, University of Texas at El Paso.

Sun, Y., Pan Z., & Shen, L. (2008). Understanding the third-person perception: Evidence from a meta-analysis. *Journal of Communication, 58*, 280-300. https://doi.org/10.1111/j.1460-2466.2008.00385.x
